# Psychological well-being among Saudi adults during the context of COVID-19 lockdown: a psychometric analysis of the 12-item General Health Questionnaire

**DOI:** 10.1186/s40359-022-01030-0

**Published:** 2022-12-27

**Authors:** Eradah O. Hamad

**Affiliations:** Department of Psychology, King Abdulaziz University, Jeddah, Saudi Arabia

**Keywords:** Psychological well-being, Mental health, The General Health Questionnaire, COVID-19 lockdown, Saudi adults, Makkah Province, Saudi Arabia

## Abstract

**Background:**

Most communities' mental health and perceptions of psychological well-being are known to be profoundly disrupted by large-scale pandemics. Despite the wide range of available screening measures, few reliable and valid screening measures exist for assessing overall psychological well-being in nonclinical populations during a health emergency situation such as the COVID-19 outbreak.

**Objective:**

This study aims to conduct a psychometric analysis of Goldberg’s 12-item General Health Questionnaire (GHQ-12) to validate its use among a sample of Saudi adults during the COVID-19 lockdown using reliability and factor analyses.

**Methods:**

A total of 473 individuals (aged 18 years and over) were recruited from the general Saudi population living in the Makkah (Western) Province of Saudi Arabia to complete the virtual format of the Arabic GHQ-12 (Ar-GHQ-12). In addition to a descriptive statistics measurement and reliability analysis, confirmatory factor analyses (CFA) were performed to examine the unidimensionality and validity of the Ar-GHQ-12.

**Results:**

In line with previous works from several cultures, the Ar-GHQ-12 with two-factor solution considered to be the best-fitting model because it fits the data better than the one-factor (unidimensional) model did, and adequate reliability indices were achieved for each factor (.83 for factor 1 and .65 for factor 2).

**Discussion:**

The Ar-GHQ-12 was determined to be suitable for assessing the overall psychological well-being of the general population in Saudi Arabia in emergency contexts and may be applied among Saudis and other Arabic-speaking populations in research and primary care settings.

## Background

### Coronavirus disease 2019 and the world’s mental health

In the beginning of 2020, the world was suddenly rampaged by a pandemic that affected the lives of all human beings around the globe, namely, induced by the new coronavirus disease 2019, or COVID-19 [1]. The pandemic has caused governments to initiate and enforce many precautionary measures. This, in turn, isolated a large proportion of the world's population and impeded their communication with family members, friends, and even coworkers. As a consequence of this social isolation, people in many societies and cultures became under great pressure and faced profound disruptions of their mental health and psychological well-being [2].

Over the coming months and years, even after the end of this pandemic, the number of people who need psychological support is expected to increase [3], not only for those who suffer from psychological disorders [4], but also for those who are under daily pressure due to the unusual conditions in which the world has come to live during total or partial home quarantine procedures [5]. The psychological distress associated with the pandemic has affected the well-being of the Saudi Arabian community, similar to the way it affected people in many countries, including students, health care professionals, and the general public. Whereas well-being is an important determinant of health and social outcomes, measures of positive and negative mental health states are needed for population-based research, especially after the pandemic.

A number of Saudi researchers have recognized the negative psychological consequences of the COVID-19 pandemic in samples from the Saudi Arabian community. For instance, Al-Ateeq et al. [6] used the Arabic version of the perceived stress scale to investigate social and demographic characteristics, psychological stress, and fears associated with the COVID-19 outbreak in a sample of 376 Saudi high school and university students, the majority of whom were female. The researchers found that more than half of the participants (55%) showed moderate levels of stress, and 30% showed high levels of stress. Aljemaiah et al. [7] also studied pandemic-related psychological distress among the Saudi Arabian public community using the Arabic version of the four-dimensional symptoms questionnaire. The study sample included 347 participants who showed high levels of stress and symptoms of depression, anxiety, and somatization. Furthermore, to measure depression and anxiety levels among health care providers during the outbreak, the study of Al-Ateeq et al. [8] used Arabic versions of the patient health questionnaire and the general anxiety scale to examine a sample of 502 health care providers working in the Ministry of Health in Saudi Arabia. The providers in the sample represented various health professions, such as administrators, nurses, physicians, specialists, technicians, and pharmacists. The results showed that more than half of the sample had depressive disorders (55.2%). Anxiety disorders were also detected in approximately half of the sample (51.49%). More validated mental health screening measures that assess both negative and positive aspects of psychological well-being are needed in the Saudi Arabian community due to the pandemic's effects on people's overall mental health. One short measure that is quite commonly used is the General Health Questionnaire (GHQ), created by Goldberg [9]. This measure was validated for use with the general population samples in various cultures [10], including the Arabian culture [11]. The examination of the scale’s validity in the Arabic literature is still limited.

### Measuring psychological well-being using the GHQ

According to the World Health Organization [12], “Mental health is a state of mental well-being that enables people to cope with the stress of life, realize their abilities, learn well, and contribute to their community.” In other words, an individual’s mental health may involve the absence of mental illness, and consequently, the presence of psychological well-being or the state of having a good life [13]. This suggests that psychological well-being is a core feature of mental health and reflects more than the absence of psychological distress (e.g., anxiety or depressive symptoms). However, mental health may exist on a complex continuum, can be experienced differently from one time to another, and may vary from one person to another and in different social and clinical settings [12]. Thus, psychological well-being should be assessed carefully by primary care providers (e.g., psychologists and family physicians) considering different aspects of a person’s mental health. The GHQ, for example, evaluates both the positive and negative mental health states of an individual [14] and can therefore be used to assist health care providers in better analyzing the public’s psychological states following the pandemic in many societies, including the Saudi Arabia.

The GHQ is a self-administered screening measure designed for use in consultation settings to assess the well-being of a person and quantify his or her risk of developing mental disorders. This measure uses simple language and can be scored easily, which makes it ideal for use in hectic clinical settings and in situations where patients require little or no assistance with completing the questions [15], including during health emergency situations such as quarantine period of the COVID-19 pandemic. The GHQ targets two areas: (a) the inability to carry out normal functions and (b) the appearance of distress. It is considered as a reliable and valid measurement and has been translated into many languages and their associated cultures, including those of Saudi Arabia (e.g., Al Kharj or the central region; [16], and Asir or the southwest region; [17]). Its original version had 60 items (GHQ-60), which were reduced to 30 (GHQ-30) and 28 (GHQ-28; [18]) for the Saudi population (see Alhamad and Al-Faris [19]) as well as 12 (GHQ-12; in the Saudi population, see El-Metwally et al. [16]) and 5 items [20]. A bimodal scale (0–0–1–1) and a 4-point Likert-type scale (0–1–2–3) are the most common scoring types used; the latter produces a more acceptable distribution of scores for parametric analysis and is recommended for the GHQ-12 to compare levels of psychological symptoms within and between samples [21]. This version of the scale has an equal number of positively and negatively worded items, and the total score of the scale ranges from 0 to 32.

The dimensionality and psychometric properties of the GHQ-12 are still a matter of debate and, therefore, have been examined in various Western and non-Western populations, including adolescents [22–24], university students [11], older adults [25, 26], urological patients [27], postpartum women [28], the general public [23], and outpatients with mental disorders [29]. As shown in Table 1, many studies have clearly demonstrated the existence of at least two subfactors in populations other than the Saudi one [30], despite it sometimes being considered unidimensional [31]. Several studies applying the two-factor model—such as in England [32], Sweden [14], and Iran [33]—used exploratory factor analyses (EFA) with either the Varimax rotation, the Oblimin rotation, or both and followed the negative and positive item division regardless of the item ordering and the factors’ names: *negative mental health, psychological distress, depression, or mental disorder* for factor I and *positive mental health or social dysfunction* for factor II. Hystad and Johnsen [30] claimed that the negative and positive item phrasing may affect the factor analysis. Furthermore, Gao et al. [29] discovered that the three subfactors identified by Graetz [22]—*anxiety and depression, social dysfunction, and loss of confidence*—fit the data better than those in other models. The model suggested by Graetz was found in most of the cultural studies employing the three-factor model regardless of the factors’ names, such as in China [29], Spain [23], and Australia [22]. In contrast to the prevalent usage of the EFA in these studies, the confirmatory factor analysis (CFA) has rarely been used (e.g., 29). The three-factor model was also examined in only one study conducted in the Saudi culture and was found to have good psychological characteristics [16]. However, like other studies from non-Western populations (e.g., [23, 24, 26]), the study conducted by El-Metwally et al. [16] used only an EFA to examine the scale’ factor structure. The resulting factor model was not compared with the scale’s general (one) mental health factor. Regarding the scale’s internal consistency, the results from previous studies were very similar indicating a satisfactory value ranging from 0.76 to 0.90.

**Table 1:** Sample studies on the reliability and factorial analyses of the GHQ-12 across cultures

Author	Sampling (culture/language)	Reliability (a)	Factor structure
*Studies of three-factor model*			
Graetz [22]	8,998 of young people aged 16–25 (Australians)	–	Anxiety (items 2,5,6,9), social dysfunction (items 1,3,4,7,8,12), and loss of confidence (items 10,11)
Daradkeh et al. [11]	157 of university students (Al-Ain/United Arab Emirates)	.86	Tension and depression (items 5,6,9,10,11), lack of enjoyment (items 1,2,7,8,12), and social dysfunction (items 3,4,11)
Gao et al. [29]	120 of outpatients with mental health problems (Chinese)	–	3-factor model of Graetz [19]
Sánchez-López and Dresch [23]	1001 subjects aged 25–65 years (Spanish)	.76	Successful coping (items 1,3,4,7,8,12), self-esteem (items 6,9,10,11), stress (items 2,5,9)
El-Metwally et al. [16]	1019 respondents aged 18 and above (Al Kharj/Saudi Arabia)	–	Social dysfunction (items 1,2.3,4,5,6), anxiety (items 7,8,9,10), and loss of confidence (items 11,12) (Positive items from 1 to 6 and negative items from 7 to 12)
*Studies of two-factor model*			
Montazeri et al. [24]	748 of young people aged 18–25 years (Persian/Iranian)	.87	Psychological distress (items 1, 3,4,7,8,10,11) and social dysfunction (items 2,5,6,7,9,12)
Doi and Minnawa [34]	1808 of general adult population aged 20 years or older (Japanese)	.83–.85	Psychological distress (items 2, 5, 6, 9, 10 and 11) and social dysfunction (items 1, 3, 4,7 and 8)
Hu et al. [32]	9204 participants, from the first wave of the British Household Panel Survey (BHPS) and 6451 participants of the Health Survey for England (HSE)	–	Mental disorder (negatively worded items) and positive mental functioning (positively worded items)
Rajabi & Sheykhshabani [33]	210 employees of a public organization Aged 25 to 57 years (Iran)	.85	Social dysfunction (positive items) and psychological distress (negative items)
Winzer et al. [14]	23,394 women, 18,274 men, aged 16–29 years (Swedish)	.84	Positive mental health (PMH) and negative mental health (NMH)
Qin et al. [26]	9692 respondents of older adults aged 60 and above (Hindi, Punjabi, Bengali and Nepali, Odia, Marathi, Malayalam, and Tamil)	.90	Depression (items 1, 8,9,10,11,12) and social dysfunction (items 2,3,4,5,6,7)

(a) Cronbach’s alpha coefficient

Considering previous findings, including those on Saudi and non-Saudi populations, which appear to confirm the GHQ-12’s multidimensionality (see Table 1), the aim of this study was to perform a psychometric analysis to assess the unidimensionality, validity, and reliability of the GHQ-12’s latent constructs in the general Saudi adult population exposed to the COVID-19 pandemic. To date, this study is considered the only one using a CFA to evaluate the unidimensionality of the GHQ-12 in the Saudi Arabian society.

## Methods

### Study design and sample

A descriptive and cross-sectional study was carried out to evaluate the psychometric properties and unidimensionality of the GHQ-12 in the Saudi general population living in Makkah Province. This province is in the western region of Saudi Arabia and includes 11governorates, such as the cities of Makkah and Jeddah, the administrative and headquarters and largest cities in the region. During the COVID-19 pandemic, an anonymous online version of the GHQ-12 and demographic questions were administered to the participants from May to July 2020 through the SurveyMonkey platform. The study population was made up exclusively of people 18 years and older with access to smartphones through a convenient snowball sampling process recruited through WhatsApp Messenger. Informed consent was obtained from the participants before they took part in the study. The study protocol was approved by the Ethics Committee of the Faculty of Arts and Humanities at King Abdulaziz University (Reference No. 4262852).

### Measurement

#### The translated GHQ-12

For the purpose of this study, the English (and publicly available) version of the GHQ-12 (e.g., Montazeri et al. [24], Qin et al. [26]) was translated into Arabic by two independent and bilingual translators using the standard forward–backward translation procedure [35]. The translated version was then back translated into English, and a committee of experts (two in mental health and two in psychometrics) reviewed both versions. After careful cultural evaluation, the committee members determined that no changes were needed for the translated items. The Arabic version of the GHQ-12 (Ar-GHQ-12) was then converted to a virtual format, and the created link was shared to facilitate its completion by the participants and reduce the risk of infection. In addition to demographic questions (e.g., sex, age, and social and job status), in the Ar-GHQ-12, the participants were asked about any symptoms and/or discomfort they had experienced recently (in the past few weeks) during the COVID-19 lockdown. Each item was graded on a 4-point scale to identify the severity of symptoms (0 = *never* [مطلقا], 1 = *rarely* [نادرا], 2 = *sometimes* [أحيانا], and 3 = *always* [دائما]).

#### Statistical analysis

IBM SPSS Statistics (Version 25) was used to analyze the sample’s demographics and the measurement items. The CFA was conducted using IBM SPSS Amos (Version 24). The CFA allowed an assessment of the Ar-GHQ-12’s unidimensionality in the Saudi general population. A CFA is often performed as a modeling technique to investigate whether a particular factor structure is consistent with the correlations or covariance of a set of observed variables. Hence, the hypothesized (latent) factor structure must be specified a priori [36]. The two models of CFA used in the analysis were based a combination of prior theory and empirical work [24] and pilot sampling, e.g., items with higher factor loadings in that factor (> 0.3). The fit indices of different models (one- and two-factor models) were also compared to consider the scores of the best fitting model.

## Results

### Descriptive statistics

The sample was made up of volunteers from the general population (n = 473), with 60.38% being female and 39.19% being male, all from Makkah (46.07%) or Jeddah (53.39%) in Makkah Province. Regarding the sample’s sociodemographic characteristics, the majority of the participants (66.31%) were between the ages of 18 and 35 years old, the second largest group comprised those between the ages of 36 and 50 years old (25.85%), whereas 4.45% were over 50 years old, and only 3.39% were under the age of 18. The study sample’s sociodemographic are shown in Table 2.

**Table 2:** Study sample characteristics (n = 473)

Variable	No.	%
*Sex*		
Male	185	39.19
Female	287	60.81
*Age*		
Less than 18	16	3.39
18–35	313	66.31
36–50	122	25.85
50 and up	21	4.45
*Social status*		
Single	226	47.88
Married	233	49.36
Divorced	10	2.12
Widowed	3	.64
*Job status*		
Student	173	36.65
Worker	210	44.49
Retired	20	4.24
No Work	69	14.62

### Reliability analysis

To analyze internal consistency, Cronbach’s alpha was calculated and values were 0.83 for factor 1 and 0.65 for factor 2, indicating satisfactory internal consistency [37]. Table 3 presents the item–scale analysis of the Ar-GHQ-12. The range of the corrected item–scale correlation was 0.60–0.38, indicating an acceptable reliability [38]. The table also shows that the standard deviations were approximately similar, suggesting no outliers existed for any of the items. The Ar-GHQ-12 item mean indicated that the degree of agreement varied between 1.75 (*rarely*) and 2.54 (*sometimes*). While the Ar-GHQ-11 (“Thinking of self as worthless”) had the highest degree of agreement (M = 2.54), the Ar-GHQ-5 (“Felt constantly under strain”) had the lowest degree of agreement (M = 1.75). The males obtained a mean score of 2.26 (SD = 0.48), and the females obtained a mean score of 2.19 (SD = 0.50). The mean difference was, however, not significant (t = 1.55, *p* > 0.05).

**Table 3:** Ar-GHQ-12 items’ descriptive and reliability statistics

Items (English/Arabic)	Item mean (SD)	Corrected item-total correlation
Ar-GHQ-1 Able to concentrate قادرعلى التركيز	2.23 (.69)	.47
Ar-GHQ-2 Loss of sleep over worry لست قادرا على النوم لساعات كافية نتيجة القلق	1.99 (.93)	.38
Ar-GHQ-3 Playing a useful role in life أقوم بعمل دور مفيد في الأشياء التي أقوم بها في الحياة مثل العمل، الدراسة، الأعمال المنزلية	2.36 (​​.74)	.38
Ar-GHQ-4 Capable of making decisions قادر على اتخاذ القرارات	2.45 (.65)	.54
Ar-GHQ-5 Felt constantly under strain كنت واقعا تحت الضغط	1.75 (.95)	.56
Ar-GHQ-6 Could not overcome difficulties لم أستطع التغلب على الصعوبات	2.15 (.80)	.60
Ar-GHQ-7 Able to enjoy day-to-day activities قادرعلى الاستمتاع بالأنشطة اليومية	2.10 (​​.82)	.59
Ar-GHQ-8 Able to face problems قادر على مواجهة المشاكل	2.28 (.69)	.56
Ar-GHQ-9 Feeling unhappy and depressed في حالة من التعاسة أو الاكتئاب	2.06 (.86)	.67
Ar-GHQ-10 Losing confidence فاقد الثقة	2.46 (.78)	.59
Ar-GHQ-11 Thinking of self as worthless التفكير بأنه لا فائدة مني	2.54 (.80)	.53
Ar-GHQ-12 Feeling reasonably happy سعيد بشكل معقول	2.27 (.70)	.57

### Goodness-of-fit statistics

**Fig. 1:**
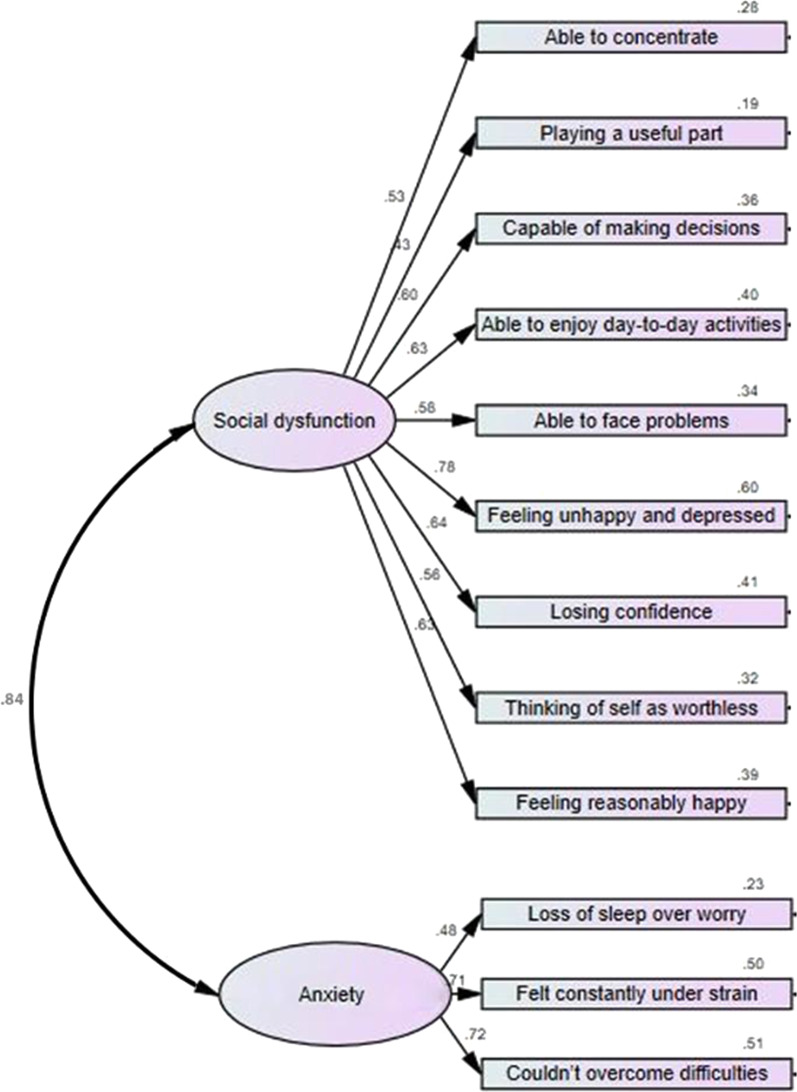
Standardized loadings of the two-factor CFA model of the Ar-GHQ-12

Comparative fit indices were used to evaluate the CFA models of the Ar-GHQ-12 using a one-factor model (a general or unidimensional mental health factor) and a two-factor model (factor 1, *personal and social dysfunction*, and factor 2, *anxiety*). The Akaike information criterion (AIC) and root mean square error of approximation (RMSEA) for the two-factor model were less than those for the one-factor model. The comparative fit index (CFI), with a value greater than 0.95, was also consistent with conventional thresholds for an acceptable fitting model. The AIC, RMSEA, and CFI values were considered as evidence of the superior fit of the two-factor model (see Table 4). Therefore, the two-factor model was considered a better (more acceptable) model fit [39]. Figure 1 displays the standardized factor loadings and between-factor correlation of the two-factor model. These loadings were correlations between the indicator variables and the latent factors (factor 1 and factor 2). The loadings ranged between 0.43 and 0.72, and the two factors were strongly correlated (r = 0.84). The strong correlation between factors suggests that even if there were two factors, distinguishing them in practice may be challenging.

**Table 4:** Goodness-of-fit of the CFA models of the Ar-GHQ-12 (n = 473)

Statistics	Model 1 (one-factor)	Model 2 (two-factors)
AIC	304.739	190.312
CFI	0.883	0.966
RMSEA	0.089	0.051

## Discussion

The GHQ-12 is a widely used screening measure for assessing overall psychological well-being and has been translated into a variety of languages and cultures. It is not, however, a tool for determining a specific diagnosis. Although the GHQ-12 items were designed as a unitary screening tool [31], efforts have been made to determine whether they have a multidimensional structure [30]. This study is one of the few studies (e.g., [16, 17]) to report data on the GHQ-12 in a sample of the Saudi population aged 18 years and older—more specifically in Makkah Province and during a health crisis. However, this study is one of the few local and cultural studies that uses a CFA framework to examine the different factor models of the translated scale (e.g., [29]). Generally speaking, the findings showed promising results and were comparable to most research findings throughout the world. The two-factor solution of this study was similar to those reported in other countries (e.g., India; [26] and Iran; [33]). However, the items loaded on these two factors were different in the current study. The two-factor model was considered the best-fitting model, and it fit the data better than the unidimensional model suggested in the GHQ-12’s original version [31]. The reliability coefficients of the Ar-GHQ-12 in the general Saudi population are 0.83 for factor 1 and 0.65 for factor 2, which is comparable to the range (0.76–0.90) found in most of the translated versions. Both the reliability and factor analyses suggest that the Ar-GHQ-12 has an acceptable number of psychometric properties and structural characteristics and can be used for measuring psychological well-being in the Saudi context among an adult population.

An important strength of the study is that the instrument demonstrates adequate reliability for this population and can be used in emergency situations such as a health crisis or in busy health care settings. Therefore, the measure is useful for making diagnostic assumptions in people seeking help and those who may require psychotherapeutic support in primary care settings. However, the cross-sectional design used in this study limits causal inferences. One limitation of this study is that the sample population was from only one region (Makkah or Western Province) in the country. Another strength is that the measure was distributed to the public on a virtual platform, thus, a large number of participants was recruited despite the quarantine restrictions. However, the virtual format may have limited contact with the participants, since no concerns or questions were raised from the participants regarding the study. Additional studies aiming to understand more about the dimensionality of the Ar-GHQ-12 in populations recruited from primary care settings and from various regions of the country are required.

In summary, the results of the CFA indicated that a two-factor solution was superior to a one-factor model. The two-factor model of the Ar-GHQ-12, therefore, has been found to be more realistic than alternative models in several populations (see Table 1), including the one presented in this study. However, the two factors are highly correlated and difficult to distinguish. Thus, this study suggests that employing this instrument as a unidimensional measure or qualitatively different constructs [30] is permissible from a practical standpoint. There is no need to consider multidimensionality unless there are particular questions arise that can only be answered only by a subset of the measure’s two components.

The availability and applicability of reliable and valid mental health evaluation instruments considering both negative and positive aspects of psychological well-being, such as the Ar-GHQ-12 used in this study, are critical for health care professionals to identify those who are at a higher risk of mental health issues following the COVID-19 pandemic, with the goal of promoting mental health interventions that can be properly planned and implemented. Future research studies with a large nonclinical-population-based sample will be valuable for evaluating the Ar-GHQ-12 variables' practical effectiveness.

## Conclusions

Examination of the unidimensionality and validity of the Ar-GHQ-12 showed that the scale has a good structural characteristics and adequate reliability, and therefore, it can be used for measuring psychological well-being in the Saudi Arabian context. Future research with a larger sample size from other provinces of the country will be helpful to assess the practical use of the Ar-GHQ-12’ dimensionality.

## Data Availability

The data set used and/or analyzed during the current study available from the corresponding author on reasonable request.
